# Impact of intra-abdominal insufflation pressure on gas leakage occurring during laparoscopy

**DOI:** 10.1007/s00464-022-09242-6

**Published:** 2022-05-03

**Authors:** Jeffrey Dalli, Tess Montminy, Makenzie Ferguson, Mohammad Faraz Khan, Kevin Nolan, Ronan A. Cahill

**Affiliations:** 1 UCD Centre for Precision Surgery, University College Dublin, Dublin, Ireland; 2 Department of Surgery, Mater Misericordiae University Hospital, Dublin, Ireland; 3 School of Mechanical and Materials Engineering, College of Engineering and Architecture, University College Dublin, Dublin, Ireland

**Keywords:** COVID, Laparoscopy, Minimally invasive surgery, Gas leaks, Occupational health

## Abstract

**Introduction:**

The advent of the COVID-19 pandemic led to recommendations aimed at minimizing the risk of gas leaks at laparoscopy. As this has continuing relevance including regarding operating room pollution, we empirically quantified carbon dioxide (CO_2_) leak jet velocity (important for particle propulsion) occurring with different instruments inserted into differing trocars repeated across a range of intra-abdominal pressures (IAPs) and modern insufflators in an experimental model.

**Method:**

Laparoscopic gas plume leak velocity (metres/second) was computationally enumerated from schlieren optical flow videography on a porcine cadaveric laparoscopic model with IAPs of 4–5, 7–8, 12–15 and 24–25 mmHg (repeated with 5 different insufflators) during simulated operative use of laparoscopic clip appliers, scissors, energy device, camera and staplers as well as Veres needle (positive control) and trocar obturator (negative control) in fresh 5 mm and 12 mm ports.

**Results:**

Close-fitting solid instruments (i.e. cameras and obturators) demonstrated slower gas leak velocities in both the 5 mm and 12 mm ports (*p* = 0.02 and less than 0.001) when compared to slimmer instruments, however, hollow instrument designs were seen to defy this pattern with the endoscopic linear stapler visibly inducing multiple rapid jests even when compared to similarly sized clip appliers (*p* = 0.03). However, on a per device basis the operating instrumentation displayed plume speeds which did not vary significantly when challenged with varying post size, IAP and a range of insufflators.

**Conclusion:**

In general, surgeon's selection of instrument, port or pressure does not usefully mitigate trocar CO_2_ leak velocity. Instead better trocar design is needed, helped by a fuller understanding of trocar valve mechanics via computational fluid dynamics informed by relevant surgical modelling.

**Supplementary Information:**

The online version contains supplementary material available at 10.1007/s00464-022-09242-6.

Although laparoscopy had seemed cemented into the 21st century’s operative modus operandi for most abdominopelvic diseases, the COVID-19 pandemic shook this paradigm to its foundations with an early and consistent concern regarding potential gas plume-associated viral aerosolization [1]. There was an immediate moratorium on its use by major surgical societies (and indeed even a stated preference for non-operative care in general) [2] and thereafter re-institution within a framework of recommendations including the use of low (“minimum”) intra-abdominal pressure (IAP) and smoke evacuation devices [3, 4]. In the crisis, this understandable and seemingly logical guidance was issued based on expert opinion alone as the existing literature based on aerosolization during laparoscopy was sparse and so such recommendations now need examination and either validation or adjustment. This is necessary, alongside the preservation of best surgical practice in any future surges or indeed pandemics, as there is now too increasing acceptance of hazards of exposure to surgical smoke (including its mutagenic and infective contents) for operating room teams. Proper hazard quantification for effective mitigation measures requires comprehensive mechanistic elucidation.

Here, we utilize previously established methodology, involving sensitive gas leak imaging in a high-fidelity surgical simulation model, to quantitatively interrogate pressure, insufflator and port-diameter-related influences on laparoscopic gas leaks occurring with common classes of instruments. A variety of available, modern insufflation systems that offer low-pressure pneumoperitoneum with high-frequency pressure sensing were utilized to assess for a class effect on the relationship between IAP and port-related gas leaks. As the main safety issue here relates to particle trajectory within laparoscopic gas leaks, rather than volume of gas leak, maximum jet leak velocities were the focus of this work. While inbuilt insufflator or add-on trocar-level smoke extraction capability to purify the composition of the pneumoperitoneum are potentially important added measures for operating room staff protection, their mitigation benefit value depends too on first understanding the base problem and the impacts of other potential influences.

## Methods

**Fig. 1:**
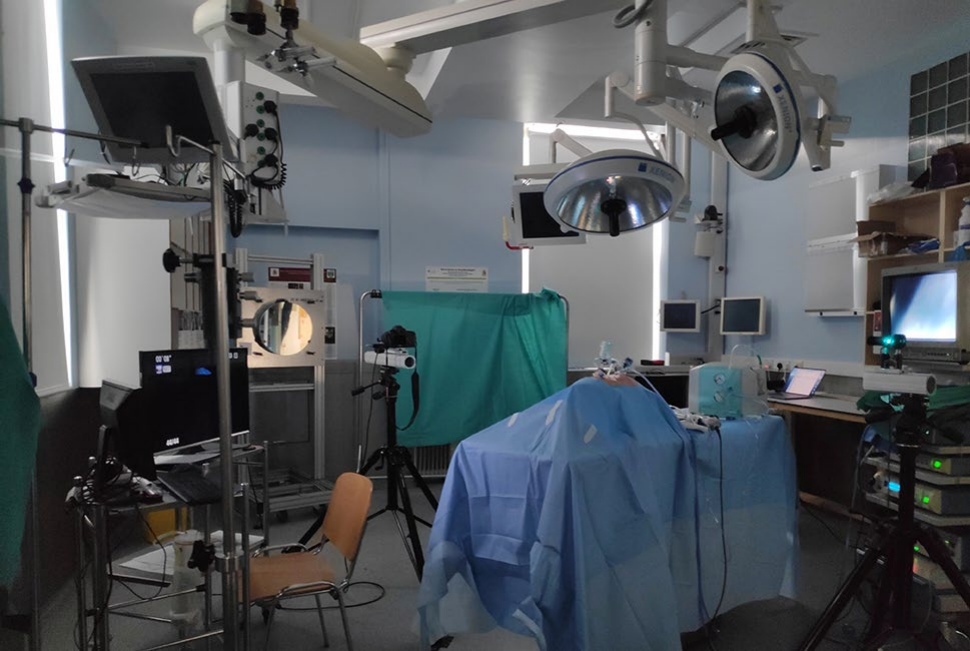
Photography of the experimental setup of a porcine cadaveric laparoscopic model in a dedicated research theatre with schlieren optical imaging system with one (of a pair) 40 cm parabolic mirror (far left) Canon 5D mk III camera with EF 100 mm f/2.8 lens (left) and laser light source (far right)

With institutional approval (AEROSOLVE, IRB 1/378/2172), a Schlieren optical flow system was deployed in a dedicated research theatre with a laparoscopic simulation model (60 kg fresh porcine cadaver) as previously reported (see Fig. 1) [5]. Schlieren imaging is a passive imaging method whereby changes in refractive index can be visualized directly and in real time enabling also high-resolution video recordings for post hoc quantitative analytics. Recording of the reflected and refracted columns of light (from two 40 cm parabolic mirrors) was carried out at 1280 × 720 pixels and at 60 frames/second via a Canon 5D mk III camera with EF 100 mm f/2.8 lens in h.264 ALL-I format. For the surgical simulation, laparoscopy was performed in the model using five different insufflators (Lexion AP 50/30 [6], Nebulae 1 Northgate Technologies [7]; EVA Palliare [8]; Dyonics Smith and Nephew; Pneumoclear Stryker [9], see Table 1) to separately provide pneumoperitoneum across a range of IAPs (Ultralow 4–5 mmHg, Low 7–8 mmHg, Medium 12–15 mmHg and High 24–25 mmHg) with the relevant company representative in attendance to ensure correct usage. Three trocars were used to perform the laparoscopy, a 10 mm Hasson port for the laparoscopic camera (Laprosurge) and both a 5 mm port (Versaone, Medtronic) and a 12 mm port (Versaport plus, Covidien 10–15 mm with the 12 mm seal) for instrumentation. When necessary, obligatory proprietary ports (e.g. Insuflow 12 mm port with Lexion AP50/30) were used as per the manufacturer’s direction. Within this setup, Schlieren imaging was used to visualize gas leaks occurring with operative instrument use (insertion, movement and removal) through the ports at different IAPs with a new set of ports used for each instrument sequence and for each insufflator. The experimental instrumentation sequence for each IAP was, in turn, a linear laparoscopic stapler (12 mm, Covidien Endo GIA) inserted into the 12 mm working port, laparoscopic clip appliers (5 mm and 11 mm, Weck AutoEndo 5 and Applied) and scissors (5 mm, Covidien Autosuture Endo shears) inserted into both the 5 mm and 12 working ports, a 10 mm laparoscopic camera scope inserted into the Hasson port, appropriate trocar obturators placed into the 5 and 12 mm ports, a laparoscopic energy device (5 mm, Harmonic Scalpel, Ethicon) inserted into both 5 mm and 12 mm trocars and finally, for positive control, a Veres needle into both the working trocars. The procedures were carried out by the same senior general surgeon with similar speed of movement to actual human laparoscopic application.

**Table 1:** Details of insufflators by brand, model and features as per the manufacturer’s literature. TAP represents True Abdominal Pressure and l/min denotes litres per minute

Brand	Lexion	Northgate Technologies	Palliare	Smith and Nephew	Stryker
Model	AP 50/30	Nebulae 1	EVA	Dyonics	Pneumoclear
Country	MN, USA	IL, USA	Galway, ROI	London, UK	Kalamazoo, USA
Smoke evac	Yes	Yes	Yes	No	YES
Heated	Yes	Yes	No	No	Yes
Humidification	Yes	Yes	No	No	YES
Max flow in l/min	50	50	40	15	50
Max pressure mmHg	25	24	15	24	15
TAP Sensing	YES	Yes	Yes	No	Yes

Post hoc video processing was performed using a custom Matlab (Mathworks, Ireland) script incorporating the Farneback optical flow algorithm to extract gas velocity in metres per second (calibrated from the 0.4 m diameter Schlieren mirror) from the pixel motion observed across sequential frames. Resulting maximum velocities, selected as the best representation of the most significant instance of the leak relevant to particle propulsion (as opposed to duration or volume), were computed in Microsoft Excel (Microsoft 365) for each experimental reading, ascribed per instrument, IAP, port and insufflator. For analysis, recordings were aggregated into three categories: 1: Camera & Obturator, 2: 5 mm instruments and 3: 11-12 mm instruments. Statistical interrogation was carried out using IBM SPSS version 27(NY, USA) with Shapiro–Wilk test for normality and appropriately applied Mann–Whitney *U*, Kruskal–Wallis and One-way ANOVA tests with further post hoc tests (Pairwise and Tukey).

## Results

**Fig. 2:**
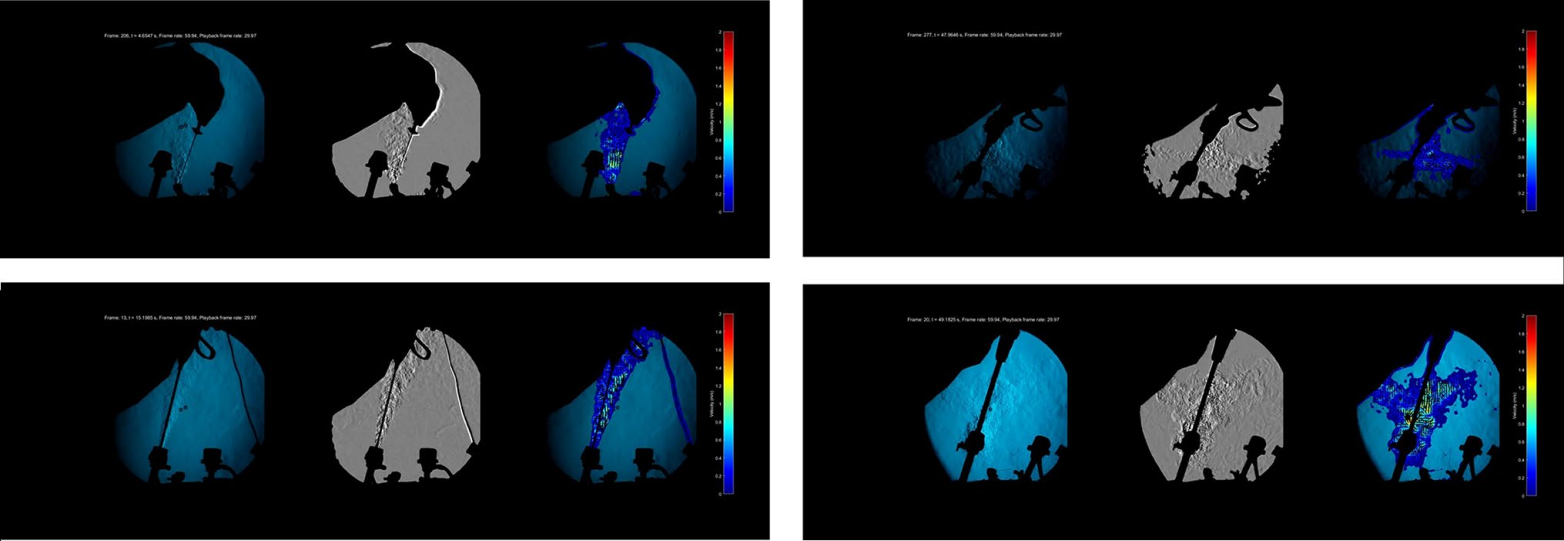
Composite collation of schlieren images in colour, grayscale and with visual representations of gas velocity using the optical flow measurements on the far right (with associated scale.). The figure shows a Veres Needle at 15 mmHg(Eva) on the Top Left; Endo GIA stapler at 15 mmHg(EVA) on the Top Right; Harmonic at 15 mmHg (Pneumoclear) on the Bottom Left and 11 mm clip applier at 25 mmHg (Nebulae 1) on the Bottom Right

Instrumentation of both trocars at all pressures during laparoscopy caused distinct plumes of gas to be released from the top of each port site. These ejected out from the trocar orifice and enveloped the instrument with the most striking leaks being seen around stapler (see Fig. 2 and Video 1). Ports also displayed gushes of gas during device manipulation during instrumentation within the valve versus a sluggish fumarole surrounding the obturator–trocar interface crevice as the obturator sat in the port, persisting despite sitting snuggly (See Video 2). Post hoc video analysis was able to consistently quantify maximum velocities (*n* = 231 individual readings) from the Schlieren video recordings (see Tables 2 and 3).

**Table 2:** Instrument categories and individual averaged maximum gas velocities in metres per second for each device at different pressure categories of IAP (Ultralow 4–5 mmHg, Low 7–8 mmHg, Medium 12–15 mmHg and High 24–25 mmHg)

Device category	Instrument	Pressure range	n	Mean ± SD Max Velocity in m/s
Category 1 (Camera and Obturator)	Camera	Ultralow	3	0.70 ± 0.80
	Camera	Low	5	0.60 ± 0.61
	Camera	Medium	6	0.44 ± 0.28
	Camera	High	3	0.41 ± 0.48
	Obturator	Ultralow	6	0.91 ± 0.51
	Obturator	Low	12	1.53 ± 0.70
	Obturator	Medium	14	1.37 ± 0.79
	Obturator	High	6	1.63 ± 1.08
Category 2 (5 mm devices)	Instrument (Scissors)	Ultralow	6	2.64 ± 1.47
	Instrument (Scissors)	Low	12	1.99 ± 0.82
	Instrument (Scissors)	Medium	14	2.42 ± 0.63
	Instrument (Scissors)	High	6	2.52 ± 0.65
	Harmonic	Ultralow	8	1.68 ± 1.67
	Harmonic	Low	11	1.98 ± 0.80
	Harmonic	Medium	13	1.95 ± 0.84
	Harmonic	High	6	1.43 ± 0.84
	Veres	Ultralow	6	1.51 ± 0.43
	Veres	Low	14	1.90 ± 0.53
	Veres	Medium	13	1.82 ± 0.54
	Veres	High	6	1.82 ± 0.27
	Clip Applier 5	Ultralow	5	1.65 ± 1.27
	Clip Applier 5	Low	7	2.00 ± 0.58
	Clip Applier 5	Medium	6	2.29 ± 0.54
	Clip Applier 5	High	3	1.68 ± 0.51
Category 3 (11–12 mm devices)	Clip Applier 11	Ultralow	5	1.90 ± 0.45
	Clip Applier 11	Low	7	1.69 ± 0.80
	Clip Applier 11	Medium	6	1.43 ± 0.30
	Clip Applier 11	High	3	2.36 ± 1.54
	Stapler GIA 12	Ultralow	3	2.17 ± 0.11
	Stapler GIA 12	Low	6	2.64 ± 1.12
	Stapler GIA 12	Medium	7	2.81 ± 0.83
	Stapler GIA 12	High	3	1.89 ± 0.65

**Table 3:** Gas leak maximum velocities (mean and standard deviation) in metres per second by instrument, port size and device category compared via the parametrically appropriate tests with post hoc analysis

Port Size	Instrument	*n*	Mean m/s ± SD	Comparison *L* < *R*	Device category	Mean m/s ± SD	Comparison *L* < *R*
5 mm	Obturator	17	1.30 ± 0.83	Kruskal–Wallis Test * p* = 0.015*	1. Camera & Obturator	1.30 ± 0.83	Mann–Whitney *U* Test * p* = 0.020*
	Instrument	19	2.32 ± 1.01	Post hoc (Pairwise)	2. 5 mm	1.91 ± 0.99	
	Harmonic	19	1.69 ± 1.22	Obturator vs Instrument * p* = 0.009*			
	Veres	19	1.74 ± 0.50				
12 mm	Camera	17	0.53 ± 0.49		1. Camera & Obturator	1.04 ± 0.79	One-Way ANOVA * p* < 0.001* Post Hoc (Tukey) Camera vs 5 mm & 11 mm * p* < 0.001* 5 mm vs 11 mm * p* = 0.827
	Obturator	21	1.46 ± 0.75	One-Way ANOVA * p* < 0.001*			
	Instrument	19	2.35 ± 0.72	Post hoc (Tukey)	2. 5 mm	2.03 ± 0.72	
	Harmonic	19	1.95 ± 0.81	Camera vs all* * p* < 0.001(Obturator * p* = 0.03)			
	Veres	20	1.86 ± 0.48	Obturator vs Stapler GIA 12 * p* < 0.001*			
	Clip applier AutoEndo 5	21	1.95 ± 0.77	Clip applier Applied 11 vs Stapler GIA 12 * p* = 0.03*			
	Clip applier Applied 11	21	1.76 ± 0.76	Obturator vs Instrument * p* = 0.003*	3. 11–12 mm	2.12 ± 0.89	
	Stapler GIA 12	19	2.51 ± 0.86				

For all comparisons the diminutive value is always ascribed first (*L* < *R*: Left small than right)

Interestingly on a per device basis, there was no significant difference in maximum gas leak velocity of operating instruments between use in the two port sizes (5 mm vs 12 mm) at any individual IAP. Juxtaposition of the broad device categories displayed a common theme with the Camera and Obturator Category resulting in slower leaks within the 12 mm port (1.04 ± 0.79 m/s) versus devices in the 5 mm and 11 mm categories (2.03 ± 0.72 m/s, 2.12 ± 0.89 m/s, *p* < 0.001) in this port. There was, however, no significant difference between instruments in either 5 mm or 11 mm categories being used in the 12 mm port at any IAP. These findings were also the same for the 5 mm port (i.e. the only significant difference was between the obturator versus the other aggregated 5 mm instruments, *p* = 0.02 and not between the working instruments themselves).

On inter-device comparison, the camera (0.53 ± 0.49 m/s) was associated with significantly slower leaks than all other devices (*p* < 0.001) including the obturator (1.46 ± 0.75 m/s *p* = 0.03) with both grouped and post hoc discrimination. The obturator itself also displayed a similar trend in the 12 mm port versus narrower instruments (2.35 ± 0.72 m/s, *p* = 0.003). Within the 5 mm port, the obturator was associated with lower gas velocities (1.30 ± 0.83 m/s) when compared to most instruments (2.32 ± 1.01 m/s *p* = 0.009) with the exception of the energy device and the Veres needle. Interestingly, instruments in the 11–12 mm group (stapler and clip appliers) had more complex gas streams separate to the width of the shaft meaning no real class effect could be seen overall. However, the linear endoscopic stapler did eject faster plumes of gas (2.51 ± 0.86 m/s) versus the 11 mm Clip applier (1.76 ± 0.76 *p* = 0.03) on Tukey post hoc interrogation. The visible leaks could be seen infiltrating the hollow design of this tool and short circuiting via numerous permeable structural loci e.g. between the gaps of the jaws.

**Fig. 3:**
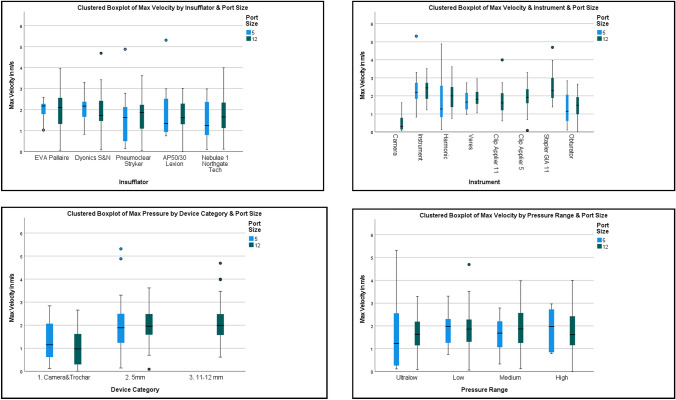
Composite diagram of box plots with error bars for mean max gas velocity in metres per second in the 5 mm(blue) and 12 mm(green) ports vs insufflators (Top left), instruments (Top Right), device categories (Bottom Right) and pressure range (Bottom Left)

Regarding differing IAPs, there was no significant difference in mean maximum velocities for all instruments being used in either the 5 mm and 12 mm ports across the different IAPs used (nor indeed was there any difference between the different insufflators themselves at any individual pressure) (see Table 4, Fig. 3).

**Table 4:** Gas leak maximum velocities (mean and standard deviation) in metres per second by port size, pressure range and insufflator compared via the parametrically appropriate tests

Port size	Mean ± SD	Comparison	Pressure range	*n*	Mean m/s ± SD	Comparison	Insufflator	*n*	Mean m/s ± SD	Comparison
5 mm	1.77 ± 0.98	Mann–Whitney *U* * p* = 0.647				Kruskal–Wallis * p* = 0.511	Palliare	13	1.96 ± 0.52	Kruskal–Wallis * p* = 0.231
			Ultralow	14	1.72 ± 1.68		Smith and Nephew	13	2.09 ± 0.70	
			Low	23	1.88 ± 0.72		Stryker	16	1.56 ± 1.23	
			Medium	25	1.67 ± 0.75		Lexion	16	1.85 ± 1.21	
			High	12	1.85 ± 0.87		Northgate	16	1.50 ± 0.94	
12 mm	1.81 ± 0.89					One-Way ANOVA * p* = 0.502	Palliare	33	1.98 ± 0.97	One-Way ANOVA * p* = 0.541
			Ultralow	28	1.64 ± 0.80		Smith and Nephew	29	1.96 ± 1.02	
			Low	51	1.81 ± 0.88		Stryker	33	1.71 ± 0.82	
			Medium	54	1.94 ± 0.92		Lexion	31	1.70 ± 0.74	
			High	24	1.72 ± 0.95		Northgate	31	1.72 ± 0.89

## Discussion

Minimally invasive surgery has revolutionized surgical practice. Its benefits in diminishing surgical trauma and improving recovery times while maintaining oncological outcomes are now widely recognized [10]. Laparoscopy, however, necessitates pressurized CO_2_ insufflation, valved ports and energy devices which in combination creates jet-streams of gas ejections (familiar to surgeons everywhere by their characteristic ‘hiss’ on instrument insertion). Such emissions may contain microparticles of biological tissue and smoke reflective of the surgical smog within the abdomen. While previous academic focus was directed towards fume-related nosocomial transmission risk of various pathogens (e.g. Hepatitis B [11], HPV [12] and HIV [13]), there is increasing awareness now too of chemical pollutants as well as the environmental impact of medical CO_2_ leakage as a greenhouse gas matching the carbon footprint of a European nation [14]. At the onset of the pandemic therefore, the knee jerk reaction was to consider laparoscopy an aerosol generating procedure and recommend to minimize its use. Despite reports identifying SARS-CoV-2 virus in peritoneal fluid [15]and faeces [16], there has thankfully been little evidence of widespread direct infection of operating rooms teams by this mechanism, helped no doubt by careful patient screening and selection as well as various other staff protection strategies including deployment of higher level respiratory protection including PAPR (Powered air purifying respirators) [17] as well as the use of air purifiers [18], underwater seals [19] and smoke evacuation devices (although none of these totally mitigate/eliminate gas or particle release [20] and therefore some inhalation risk remains). Relatively little attention has been directed towards mitigating leaks at their source from theatre disposables such as ports and instrument devices. To futureproof and safeguard surgical practice, this needs address.

Since the onset of the pandemic, we have utilized thermographic [21], optical (lasers) [22] and particle counters [23] to demonstrate and quantify gas and scintillant smoke particles as they are expelled up into the breathing zone of the operating room team during both abdominal surgery and endo-anal surgery [23]. These plumes have been quantified to achieve velocities of 5 m/s (metres/second) [5] across multiple particle sizes (0.3–10 μm) including within the aerosol (< 1 μm) phase and persist despite positive room ventilation [22]. Our established cadaveric porcine schlieren setup [5] with post hoc computational quantification has now allowed the statistical interrogation and dissection of the relative contribution of port(trocar bore):instrument diameter ratio as well as the impact of IAP and its modulation which have been postulated as potentially important factors in mitigation (in tandem with increasing evidence of low IAP association with improved patient outcomes) [24]. In this, we have found that surgeon selection of instrument, port or pressure does not usefully mitigate trocar CO_2_ leak velocity.

Our choice of maximum velocity as the quantifiable metric focuses on the peak propulsive energy of the gas leaks and thus the reach of particles as this is of most relevance to pollution into the breathing zone of the operating room team rather than the overall displaced volume of CO_2_. We have previously calculated temporal-volumetric flow rate in litres per second and thus estimated Reynolds number as measure of flow characteristic (laminar vs turbulent), for example anecdotally showing that instrument insertion results in a more copious and more turbulent jet than withdrawal [5]. Neither is perfect as trocar gas leaks take place at the valve-instrument slit aperture and valves are known to deform, fatigue, behave differently at different IAPs. Volumetric calculations must also take into consideration the other internal dimensions of the port which may vary with movement-related valve distortion. For example, a lower velocity leak may be taking place across a larger orifice resulting in a larger volume of gas effluvium, albeit projecting for a shorter distance or with less momentum to disperse or aerosolize larger particles. Nonetheless, the use of gas velocity in m/s in newly opened and instrumented ports gives an indicative measure of clinical value although this should not oversimplify the nuances of port design.

Our results indicate that only the largest instruments that nearly fully occlude the internal port diameter have any association with significant diminution of gas leak velocity (and so also overall ejected volume). However, instrument designs which permit gas flow into and through negate any such benefits (as seen with the Endo GIA stapler). Our data show that for narrow instruments there are no realistic benefit to using a smaller port, and while port incisions should be kept to a minimum to avoid peri-port leaks as per guidance [4] and operative trauma, smaller ports do not result in slower ejected gas. Thus, one should not sacrifice larger access if needed (and in fact, starting off small and then intra-operatively upsizing ports may in fact deleteriously result in unnecessary abdominal wall gas leaks). The data also fail to show significantly slower plumes at lower pressures. This is possibly because port valves have been designed to close at particular pressures and the lower IAP in fact fails to recruit the valve leaflets to effectively stop gas backflow out of the cavity. While reduced IAP may offer advantages for patient recovery [24], when one considers gas leaks it is important to balance this potential positive with the technical challenges potentially conferred by low IAP (especially diminished visualization in patients with higher BMIs and prolonged operative times) that may idiosyncratically increase the overall frequency of trocar leaks jeopardizing staff occupational safety.

A limitation of this study is that only one single type of trocar was used and of course there are very many different makes and models of trocars in regular use around the world. Furthermore, despite robust technical quality assurance measures being in place with all major commercial manufacturers, a single individual trocar may not be necessarily representative of a large sample of even the same brand. Nonetheless, the specific trocar used in this study, intended to investigate the relative importance of pneumoperitoneum pressure and insufflator performance as well as instrument:port diameter matching, is a very common port from a major global supplier [25]. Furthermore, while some trocars do seem to perform better than others regarding gas leakage [26, 27], this study examining effects of pressure and bore diameter on leak magnitude of course needed to use a trocar that leaks some gas in order to usefully test its hypotheses. As trocar type is for sure a factor in gas leaks, examination of the effects of any single variable (here in sequence insufflator type, pneumoperitoneal pressure and instrument:trocar matching) needs consistency regarding all other variables and so the same trocar was used across the experiment (with a new trocar each time) and considerable preliminary work had been done to develop and validate the model used in all its aspects. With respect to optimum trocar type determination (a topic not addressed here), trocars have been seen to behave differently regarding gas leaks during static baseline versus dynamic instrumentation and indeed, supported by the data in this study and others [26, 28] it seems variations in instruments between manufacturers impact leak volume more significantly. However, even studies for the specific purpose of trocar performance examination (that therefore examine many trocars under standardized conditions) do not include all existing brands and indeed the large variation of valve types and geometries complicates conclusive statistical inferences comparing many different trocars. All this means that this is a complex area to fully understand and enable proper standards. While of course surgeons and surgical care need simplicity and clarity regarding technique and technology guidance, such advice needs to be true and empirically provable.

In conclusion, gas leakage does not seem to be mitigated through the use of smaller ports for fitting devices for the majority of working instruments or indeed through lower pressure laparoscopy. This pandemic has exposed our lack of attention to the rather complex aspect of medical devices in the overall operative habitat and the need for future design priorities to surpass immediate blueprinting, manufacturing, and testing and provide sound computational models reflecting the fluid dynamic interactions of their devices with the general operating room environment where they are deployed. This should better inform operating protocols, guidelines, theatre airflow configuration and infection control protocols to fortify theatre team safety and operative room productivity as we sail the waves of this and future pandemics.

## Supplementary Information

Below is the link to the electronic supplementary material.

Composite collation of schlieren videos in colour, grayscale and with visual representations of gas velocity using the optical flow measurements on the far right of simulated instrumentation of a laparoscopic linear stapler Endo GIA 12 mm at an intra-abdominal pressure of 12 mmHg utilizing the Eva insufflator (MP4 87059 kb)

Schlieren video in colour illustrating port leaks (5 and 12 mm) while inserting the obturator in the respective 5 and 12 mm ports at 8 mmHg IAP with the Nebulae 1 insufflator (AVI 2357 kb)
